# Coupling all-atom molecular dynamics simulations of ions in water with Brownian dynamics

**DOI:** 10.1098/rspa.2015.0556

**Published:** 2016-02

**Authors:** Radek Erban

**Affiliations:** Mathematical Institute, University of Oxford, Radcliffe Observatory Quarter, Woodstock Road, Oxford OX2 6GG, UK

**Keywords:** multiscale modelling, molecular dynamics, Brownian dynamics

## Abstract

Molecular dynamics (MD) simulations of ions (K^+^, Na^+^, Ca^2+^ and Cl^−^) in aqueous solutions are investigated. Water is described using the SPC/E model. A stochastic coarse-grained description for ion behaviour is presented and parametrized using MD simulations. It is given as a system of coupled stochastic and ordinary differential equations, describing the ion position, velocity and acceleration. The stochastic coarse-grained model provides an intermediate description between all-atom MD simulations and Brownian dynamics (BD) models. It is used to develop a multiscale method which uses all-atom MD simulations in parts of the computational domain and (less detailed) BD simulations in the remainder of the domain.

## 1. Introduction

Molecular dynamics (MD) simulations of ions in aqueous solutions are limited to modelling processes in relatively small domains containing (only) several thousands of water molecules [1,2]. Ions play important physiological functions in living cells which typically consist of 10^10^ to 10^12^ water molecules. In particular, processes which include transport of ions between different parts of a cell cannot be simulated using standard all-atom MD approaches. Coarser models are instead used in applications. Examples include Brownian dynamics (BD) simulations [3] and mean-field Poisson–Nernst–Planck equations [4]. In BD methods, individual trajectories of ions are described using

1.1 $$dX_{i}=\sqrt{2D}\;dW_{i},\;i=1,2,3,$$

where **X**=(*X*_1_,*X*_2_,*X*_3_) is the position of the ion, *D* is its diffusion constant and *W*_*i*_, *i*=1,2,3, are three independent Wiener processes [5]. BD description (1.1) does not explicitly include solvent molecules in the simulation. Moreover, in applications, equation (1.1) can be discretized using a (nanosecond) timestep which is much larger than the typical timestep of MD simulations (femtosecond) [6]. This makes BD less computationally intensive than the corresponding MD simulations.

Longer timesteps of BD simulations enable efficient simulations of ion transport between different parts of the cell, but they limit the level of detail which can be incorporated into the model. For example, intracellular calcium is regulated by the release of Ca^2+^ ions from the endoplasmic reticulum via inositol-4,5-triphosphate receptor (IP_3_R) channels. BD models in the literature use equation (1.1) to describe trajectories of calcium ions [3,7]. The conformational changes between the open and closed states of IP_3_R channels are controlled by the binding of Ca^2+^ to activating and inhibitory binding sites. BD models postulate that binding of an ion occurs with some probability whenever the distance between the ion and an empty site is less than the specific distance, the so-called reaction radius [8,9]. Although details of the binding process are known [10,11], they cannot be incorporated into coarse BD models of calcium dynamics, because equation (1.1) does not correctly describe short-time behaviour of ion dynamics.

The calcium-induced calcium release through IP_3_R channels is an example of a multiscale dynamical problem where MD simulations are important only in certain parts of the computational domain (close to an IP_3_R channel), while in the remainder of the domain a coarser, less detailed, BD method could be used (to describe trajectories of ions). Such multiscale problems cannot be simulated using MD methods, but there is potential to design multiscale computational methods which compute the desired information with an MD-level of resolution by using MD and BD models in different parts of the computational domain [12].

In [12], three relatively simple and analytically tractable MD models are studied (describing heat bath molecules as point particles) with the aim of developing and analysing multiscale methods which use MD simulations in parts of the computational domain and less detailed BD simulations in the remainder of the domain. In this follow-up paper, the same question is investigated in all-atom MD simulations which use the SPC/E model of water molecules. In order to couple MD and BD simulations, we need to first show that the MD model is in a suitable limit described by a stochastic model which does not explicitly take into account heat bath (water) molecules. In [12], this coarser description was given in terms of Langevin dynamics. Considering all-atom MD simulations, the coarser stochastic model of an ion is more complicated than Langevin dynamics. In this paper, it will be given by

1.2 $$\begin{array}{rl} dX_{i} & =V_{i}\;dt, \end{array}$$

1.3 $$\begin{array}{rl} dV_{i} & =U_{i}\;dt, \end{array}$$

1.4 $$\begin{array}{rl} dU_{i} & =(-\eta_{1}V_{i}+Z_{i})\;dt \end{array}$$

1.5 $$\begin{array}{rl} \text{and}\;\;dZ_{i} & =-(\eta_{2}Z_{i}+\eta_{3}U_{i})\;dt+\eta_{4}\;dW_{i},\;i=1,2,3, \end{array}$$

where **X**≡(*X*_1_,*X*_2_,*X*_3_) is the position of the ion, **V**≡(*V*_1_,*V*_2_,*V*_3_) is its velocity, **U**≡(*U*_1_,*U*_2_,*U*_3_) is its acceleration, **Z**≡(*Z*_1_,*Z*_2_,*Z*_3_) is an auxiliary variable, *d***W**≡(*dW*_1_,*dW*_2_,*dW*_3_) is white noise and *η*_*j*_, *j*=1,2,3,4, are parameters. These parameters will be chosen according to all-atom MD simulations as discussed in §3. In §4, we show that (1.2)–(1.5) provides a good approximation of ion behaviour. In §5, we further analyse the system (1.2)–(1.5) and show how parameters *η*_*j*_, *j*=1,2,3,4, can be connected with diffusion constant *D* used in the BD model (1.1).

The coarse-grained model (1.2)–(1.5) is used as an intermediate model between the all-atom MD model and BD description (1.1). In §5, we show how it can be coupled with the BD model which uses a much larger timestep than the MD model. In §6, the coarse-grained model (1.2)–(1.5) is coupled with all-atom MD simulations. We then show that all-atom MD models of ions can be coupled with BD description (1.1) using the intermediate coarse-grained model (1.2)–(1.5). In §7, we introduce a hierarchy of stochastic coarse-grained models which generalize the coarse-grained model (1.2)–(1.5) and can be used to fit additional properties of all-atom MD. This generalization is formulated in terms of fictitious particle models [13] which are themselves based on earlier work in non-equilibrium statistical physics [14–17]. In particular, we show that the dynamics of the intermediate coarse-grained model (1.2)–(1.5) can be equivalently described by an appropriately formulated fictitious particle model [13]. We conclude by discussing related methods developed in the literature in §8.

## 2. Molecular dynamics simulations of ions in SPC/E water

There have been several MD models of liquid water developed in the literature. The simplest models (e.g., SPC [18], SPC/E [19] and TIP3P [20]) include three sites in total, two hydrogen atoms and an oxygen atom. More complicated water models include four, five or six sites [21,22]. In this paper, we use the three-site SPC/E model of water which was previously used for MD simulations of ions in aqueous solutions [23,1]. In the SPC/E model, the charges (*q*_h_ =0.4238 e) on hydrogen sites are at 1 Å from the Lennard–Jones centre at the oxygen site which has negative charge *q*_o_=−0.8476 e. The HOH angle is 109.47°. We use the RATTLE algorithm [24] to satisfy constraints between atoms of the same water molecule.

We investigate four ions (K^+^, Na^+^, Ca^2+^ and Cl^−^) at 25°C using MD parameters presented in [23]. Let us consider a water molecule and let us denote by *r*_*i*0_ (respectively, *r*_*i*1_ and *r*_*i*2_) the distance between the ion and the oxygen site (respectively, the first and second hydrogen sites). The pair potential between the water molecule and the ion is then given by [1,23],

2.1 $$A_{\mathrm{io}}{\left(\frac{1}{r_{\mathrm{io}}}\right)}^{12}-B_{\mathrm{io}}{\left(\frac{1}{r_{\mathrm{io}}}\right)}^{6}+k_{e}\frac{q_{i}q_{o}}{r_{i0}}+k_{e}\frac{q_{i}q_{h}}{r_{i1}}+k_{e}\frac{q_{i}q_{h}}{r_{i2}},$$

where *A*_io_ and *B*_io_ are Lennard–Jones parameters between the oxygen on the water molecule and the ion, *k*_*e*_ is Coulomb's constant and *q*_*i*_ is the charge on the ion. The values of parameters are given for four ions considered in table 1. We express mass in daltons (Da), length in ångströms (Å) and time in picoseconds (ps), consistently in the whole paper. Using these units, the parameters of the Lennard–Jones potential between the oxygen sites on two SPC/E water molecules are *A*_oo_=2.6334×10^8^ Da Å^14^ ps^−2^ and *B*_oo_=2.6171×10^5^ Da Å^8^ ps^−2^.

**Table 1.** Parameters of all-atom MD simulations of ions.

ion	*A*_io_ [Da Å^14^ ps^−2^]	*B*_io_ [Da Å^8^ ps^−2^]	*q*_*i*_ [e]	*M* [Da]
K^+^	2.8973×10^8^	2.4587×10^5^	+1	39.0983
Na^+^	6.6813×10^7^	1.1807×10^5^	+1	22.9898
Ca^2+^	1.1961×10^8^	1.5797×10^5^	+2	40.078
Cl^−^	1.8038×10^9^	6.1347×10^5^	−1	35.453

We consider a cube of side *L*=24.83 Å containing 511 water molecules and 1 ion, i.e. we have 8^3^=512 molecules in our simulation box. In the following section, we use standard NVT simulations where the temperature is controlled using Nosé–Hoover thermostat [25,26] and the number of particles is kept constant by implementing periodic boundary conditions. In particular, we assume that our simulation box is surrounded by periodic copies of itself. Then the long-range (Coulombic) interactions can be computed using several different approaches, including the Ewald summation or the reaction field method [27,28]. We use the cut-off sphere of radius *L*/2 and the reaction field correction as implemented in [1]. This approach is more suitable for multiscale methods (studied later in §6) than the Ewald summation technique. The MD timestep is for all MD simulations in this paper chosen as Δ*t*=10^−3^ ps =1 fs.

## 3. Parametrization of the coarse-grained model of ion

In MD simulations, an ion is described by its position **X**≡(*X*_1_,*X*_2_,*X*_3_) and velocity **V**≡(*V*_1_,*V*_2_,*V*_3_) which evolve according to

3.1 $$dX_{i}=V_{i}\;dt$$

and

3.2 $$M\;dV_{i}=F_{i}\;dt,\;i=1,2,3,$$

where *M* is the mass of the ion (given in table 1) and **F**≡(*F*_1_,*F*_2_,*F*_3_) is the force acting on the ion. We use all-atom MD simulations as described in §2 to estimate diffusion coefficient *D* and second moments of *V*_*i*_ and *U*_*i*_=*F*_*i*_/*M*, *i*=1,2,3. They are given for four ions considered in table 2. To estimate $\langle U_{i}^{2}\rangle ,$ we calculate the average force in the *i*th direction $\langle F_{i}^{2}\rangle ,$ where 〈⋅〉 denotes an average over sufficiently large time interval (nanosecond) of MD simulations. Taking into account the symmetry of the problem, we estimate $\langle U_{i}^{2}\rangle =\langle F_{i}^{2}\rangle /M^{2}$ as the average over all three dimensions

$$\frac{\left\langle U_{1}^{2}\rangle +\langle U_{2}^{2}\rangle +\langle U_{3}^{2}\right\rangle }{3}.$$

This value is reported in table 2. In the same way, the reported values of $\left\langle V_{i}^{2}\right\rangle$ are computed as averages over all three dimensions. Diffusion constant *D* can be estimated by calculating mean square displacements or velocity autocorrelation functions. In table 2, we report the values of *D* which were estimated in [1] by calculating mean square displacements.

**Table 2.** Average values obtained by all-atom MD simulations of ions.

ion	*D* [ Å^2^ ps^−1^]	$\left\langle V_{i}^{2}\right\rangle$ [ Å^2^ ps^−2^]	$\left\langle U_{i}^{2}\right\rangle$ [ Å^2^ ps^−4^]	$\left\langle Z_{i}^{2}\right\rangle$ [ Å^2^ ps^−6^]
K^+^	0.183	6.32	4.86×10^3^	1.65×10^7^
Na^+^	0.128	10.8	2.21×10^4^	8.88×10^7^
Ca^2+^	0.053	6.18	1.87×10^4^	9.23×10^7^
Cl^−^	0.177	6.98	6.56×10^3^	2.97×10^7^

Let us consider the coarse-grained model (1.2)–(1.5) and let 〈⋅〉 denotes an average over many realizations of a stochastic process. Multiplying equations (1.3) and (1.4) by *V*_*i*_ and *U*_*i*_, respectively, we obtain the following ordinary differential equations (ODEs) for second moments:

3.3 $$\frac{d}{dt}\langle V_{i}^{2}\rangle =2\langle U_{i}V_{i}\rangle$$

and

3.4 $$\frac{d}{dt}\langle U_{i}^{2}\rangle =-2\eta_{1}\langle U_{i}V_{i}\rangle +2\langle U_{i}Z_{i}\rangle .$$

Consequently, we obtain that 〈*U*_*i*_*V*_*i*_〉=0 and 〈*U*_*i*_*Z*_*i*_〉=0 at steady state. Multiplying equations (1.3)–(1.5) by *V*_*i*_, *U*_*i*_ and *Z*_*i*_, respectively, and taking averages, we obtain

3.5 $$\frac{d}{dt}\langle U_{i}V_{i}\rangle =\langle U_{i}^{2}\rangle -\eta_{1}\langle V_{i}^{2}\rangle +\langle V_{i}Z_{i}\rangle$$

and

3.6 $$\frac{d}{dt}\langle V_{i}Z_{i}\rangle =\langle U_{i}Z_{i}\rangle -\eta_{2}\langle V_{i}Z_{i}\rangle -\eta_{3}\langle U_{i}V_{i}\rangle .$$

Using 〈*U*_*i*_*V*_*i*_〉=0 and 〈*U*_*i*_*Z*_*i*_〉=0, we obtain that 〈*V*_*i*_*Z*_*i*_〉=0 at steady state and

3.7 $$\eta_{1}=\frac{\left\langle U_{i}^{2}\right\rangle }{\left\langle V_{i}^{2}\right\rangle }.$$

This equation is used in table 3 to estimate *η*_1_ using the MD averages $\left\langle U_{i}^{2}\right\rangle$ and $\left\langle V_{i}^{2}\right\rangle$ which are given in table 2. Since we know the value of *η*_1_, we can also estimate the value of $\left\langle Z_{i}^{2}\right\rangle$ by calculating the second moment of

3.8 $$\langle Z_{i}^{2}\rangle \approx \left\langle {\left(\frac{U_{i}(t+\Delta t)-U_{i}(t)}{\Delta t}+\eta_{1}V_{i}\right)}^{2}\right\rangle .$$

This value is reported in the last column of table 2. Multiplying equation (1.4) by *Z*_*i*_ and equation (1.5) by *U*_*i*_, we obtain

3.9 $$\frac{d}{dt}\langle U_{i}Z_{i}\rangle =\langle Z_{i}^{2}\rangle -\eta_{1}\langle V_{i}Z_{i}\rangle -\eta_{2}\langle U_{i}Z_{i}\rangle -\eta_{3}\langle U_{i}^{2}\rangle .$$

Using 〈*U*_*i*_*Z*_*i*_〉=0 and 〈*V*_*i*_*Z*_*i*_〉=0, we obtain at steady state

3.10 $$\eta_{3}=\frac{\left\langle Z_{i}^{2}\right\rangle }{\left\langle U_{i}^{2}\right\rangle }.$$

**Table 3.** Values of *η*_*j*_, *j*=1,2,3,4, estimated by (3.7), (3.10), (3.15) and (3.17) using theresults of all-atom MD simulations reported in table 2.

ion	*η*_1_ [ps^−2^]	*η*_2_ [ps^−1^]	*η*_3_ [ps^−2^]	*η*_4_ [ Å ps^−7/2^]
K^+^	768.7	152.5	3.393×10^3^	7.094×10^4^
Na^+^	2.044×10^3^	166.1	4.020×10^3^	1.717×10^5^
Ca^2+^	3.026×10^3^	190.2	4.933×10^3^	1.874×10^5^
Cl^−^	940.0	189.7	4.524×10^3^	1.061×10^5^

Multiplying equation (1.2) by *X*_*i*_, *V*_*i*_, *U*_*i*_ and *Z*_*i*_ and equations (1.3)–(1.5) by *X*_*i*_ and taking averages, we obtain the following system of ODEs for second moments:

3.11 $$\begin{array}{rl} \frac{d}{dt}\langle X_{i}^{2}\rangle & =2\langle X_{i}V_{i}\rangle , \end{array}$$

3.12 $$\begin{array}{rl} \frac{d}{dt}\langle X_{i}V_{i}\rangle & =\langle V_{i}^{2}\rangle +\langle X_{i}U_{i}\rangle , \end{array}$$

3.13 $$\begin{array}{rl} \frac{d}{dt}\langle X_{i}U_{i}\rangle & =\langle U_{i}V_{i}\rangle -\eta_{1}\langle X_{i}V_{i}\rangle +\langle X_{i}Z_{i}\rangle \end{array}$$

3.14 $$\begin{array}{rl} \text{and}\;\;\frac{d}{dt}\langle X_{i}Z_{i}\rangle & =\langle V_{i}Z_{i}\rangle -\eta_{2}\langle X_{i}Z_{i}\rangle -\eta_{3}\langle X_{i}U_{i}\rangle . \end{array}$$

Consequently, at equilibrium, we obtain 〈*X*_*i*_*V*_*i*_〉=*D*, $\langle X_{i}U_{i}\rangle =-\langle V_{i}^{2}\rangle ,$ 〈*X*_*i*_*Z*_*i*_〉=*η*_1_*D* and

$$\eta_{2}=-\frac{\eta_{3}\langle X_{i}U_{i}\rangle }{\left\langle X_{i}Z_{i}\right\rangle }=\frac{\eta_{3}\langle V_{i}^{2}\rangle }{\eta_{1}D}.$$

Using (3.7) and (3.10), we have

3.15 $$\eta_{2}=\frac{\left\langle Z_{i}^{2}\right\rangle }{D}{\left(\frac{\left\langle V_{i}^{2}\right\rangle }{\left\langle U_{i}^{2}\right\rangle }\right)}^{2}.$$

Finally, multiplying equation (1.5) by *Z*_*i*_, we obtain

3.16 $$\frac{d}{dt}\langle Z_{i}^{2}\rangle =-2\eta_{2}\langle Z_{i}^{2}\rangle -2\eta_{3}\langle U_{i}Z_{i}\rangle +\eta_{4}^{2}.$$

Consequently, we obtain at steady state

$$\eta_{4}^{2}=2\eta_{2}\langle Z_{i}^{2}\rangle .$$

Using (3.15), we get

3.17 $$\eta_{4}=\sqrt{\frac{2}{D}}\frac{\left\langle V_{i}^{2}\rangle \langle Z_{i}^{2}\right\rangle }{\left\langle U_{i}^{2}\right\rangle }.$$

The values calculated by (3.10), (3.15) and (3.17) are presented in table 3.

## 4. Accuracy of the coarse-grained model of ion

The coarse-grained model (1.2)–(1.5) has four parameters *η*_*j*_, *j*=1,2,3,4. To parametrize this model, we have used four quantities estimated from detailed MD simulations, diffusion constant *D* and steady-state values of $\langle V_{i}^{2}\rangle ,$ $\left\langle U_{i}^{2}\right\rangle$ and $\left\langle Z_{i}^{2}\right\rangle$. In particular, the coarse-grained model (1.2)–(1.5) will give the same values of these four quantities, including the value of diffusion constant *D* which is the sole parameter of the BD model (1.1). In this section, we explain why the coarse-grained description given by (1.2)–(1.5) can be used as an intermediate model to couple BD and MD models.

We begin by illustrating why Langevin dynamics (which is used in [12] for a similar multiscale problem) is not suitable for all-atom MD simulations studied in this paper. In [12], a few (heavy) particles with mass *M* and radius *R* are considered in the heat bath consisting of a large number of light point particles with masses *m*≪*M*. The collisions of particles are without friction, which means that post-collision velocities can be computed using the conservation of momentum and energy. In this case, it can be shown that the description of heavy particles converges in an appropriate limit to Brownian motion given by equation (1.1). One can also show that the model converges to Langevin dynamics (in the limit *m*/*M*→0) [29–31]:

4.1 $$dX_{i}=V_{i}\;dt$$

and

4.2 $$dV_{i}=-\gamma V_{i}\;dt+\gamma \sqrt{2D}\;dW_{i},\;i=1,2,3,$$

where **X**≡(*X*_1_,*X*_2_,*X*_3_) is the position of a diffusing molecule, **V**≡(*V*_1_,*V*_2_,*V*_3_) is its velocity, *D* is the diffusion coefficient and *γ* is the friction coefficient. In [12], Langevin dynamics (4.1)–(4.2) is used as an intermediate model which enables the implementation of BD description (1.1) and the original detailed model in different parts of the computational domain.

Langevin dynamics (4.1)–(4.2) describes a diffusing particle in terms of its position and velocity, i.e. it uses the same independent variables for the description of an ion as the MD model (3.1)–(3.2). Langevin dynamics can be further reduced to BD model (1.1) in the overdamped limit γ→∞. However, it cannot be used as an intermediate model between BD and all-atom MD simulations considered in this paper, because it does not correctly describe the ion behaviour at times comparable with the MD timestep Δ*t*. To illustrate this, let us parametrize Langevin dynamics (4.1)–(4.2) using diffusion constant *D* and the second velocity moment $\left\langle V_{i}^{2}\right\rangle$ estimated from all-atom MD simulations. To get the same second moment of velocity, Langevin dynamics requires that we choose

4.3 $$\gamma =\frac{\left\langle V_{i}^{2}\right\rangle }{D}.$$

Discretizing equation (4.2), the ion acceleration during one timestep is

4.4 $$\frac{V_{i}(t+\Delta t)-V_{i}(t)}{\Delta t}=-\gamma V_{i}(t)+\gamma \sqrt{\frac{2D}{\Delta t}}\;\xi_{i},$$

where (*ξ*_1_,*ξ*_2_,*ξ*_3_) is a vector of normally distributed random numbers with zero mean and unit variance. Using (4.3), the second moment of the right-hand side of (4.4) is

4.5 $$\gamma^{2}\left(\langle V_{i}^{2}\rangle +\frac{2D}{\Delta t}\right)=\frac{{\left(\langle V_{i}^{2}\rangle \right)}^{3}}{D^{2}}+\frac{2{\left(\langle V_{i}^{2}\rangle \right)}^{2}}{D\;\Delta t}.$$

Using the MD values of *D* and $\left\langle V_{i}^{2}\right\rangle$ for K^+^ which are given in table 2 and using MD timestep Δ*t*=10^−3^ ps, we obtain that the second moment (4.5) is equal to 4.44×10^5^ Å^2^ ps^−4^. On the other hand, $\left\langle U_{i}^{2}\right\rangle$ estimated from all-atom MD simulations and given in table 2 is 4.86×10^3^ Å^2^ ps^−4^ which is one hundred times smaller. The main reason for this discrepancy is that Langevin dynamics postulates that the random force in equation (4.2) acting on the particle at time *t* is not correlated to the random force acting on the particle at time *t*+Δ*t*. However, this is not true for all-atom MD simulations where random force terms at subsequent timesteps are highly correlated.

Since Langevin dynamics is not suitable for coupling MD and BD models, we need to introduce a stochastic model of ion behaviour which is more complicated than Langevin dynamics. The coarse-grained model (1.2)–(1.5) studied in this paper is a relatively simple example of such a model. Its parametrization, discussed in §3, guarantees that the coarse-grained model (1.2)–(1.5) well approximates all-atom MD simulations at equilibrium. They both have the same value of diffusion constant *D* and steady-state values of $\langle V_{i}^{2}\rangle ,$ $\left\langle U_{i}^{2}\right\rangle$ and $\left\langle Z_{i}^{2}\right\rangle$. Next, we show that the coarse-grained model (1.2)–(1.5) also compares well with all-atom MD simulations at shorter timescales. We consider the rate of change of acceleration (jerk or the scaled derivative of force). We define the average jerk as a function of current velocity and acceleration of the ion:

4.6 $$J(v,u)=\lim_{\tau \to 0}\frac{\left\langle U_{i}(t+\tau )-u\;|\;V_{i}(t)=v,U_{i}(t)=u\right\rangle }{\tau }.$$

To estimate *J*(*v*,*u*) from all-atom MD simulations, we calculate the rate of change of acceleration during each MD timestep

**Figure 1.**
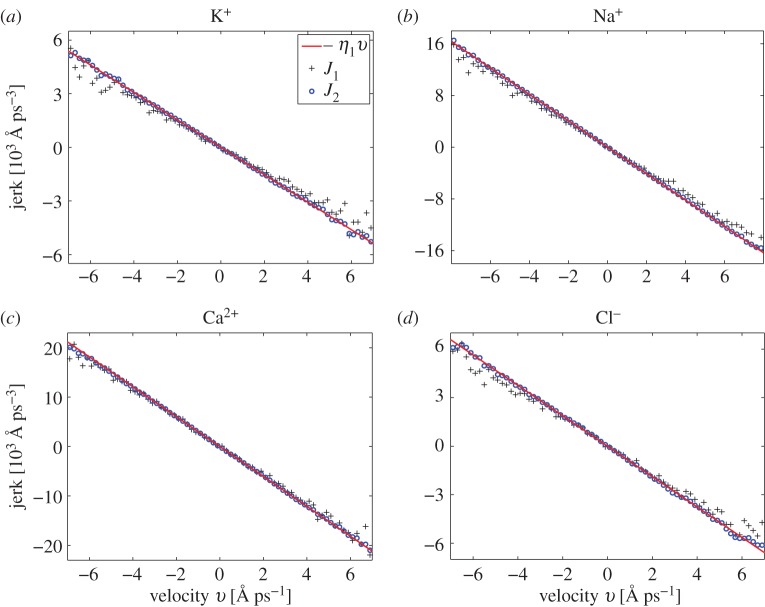
Comparison of the rate of change of acceleration (jerk) computed by all-atom MD simulations and by the coarse-grained model (1.2)–(1.5). MD results are visualized using functions *J*_1_(*v*) (black crosses) and *J*_2_(*v*) (blue circles) defined by equation (4.8). The coarse-grained model result is given by formula (4.10) (red solid line). We consider (*a*) K^+^ ion; (*b*) Na^+^ ion; (*c*) Ca^2+^ ion and (*d*) Cl^−^ ion. Parameters are given in tables 1 and 3.

4.7 $$J(v,u)\approx \frac{\left\langle U_{i}(t+\Delta t)-u\;|\;V_{i}(t)=v,U_{i}(t)=u\right\rangle }{\Delta t},$$

i.e. we run a long (nanosecond) MD simulation, calculate the values of (*U*_*i*_(*t*+Δ*t*)−*U*_*i*_(*t*))/Δ*t* during every timestep and record their average in two-variable array *J*(*v*,*u*) indexed by binned values of *V*_*i*_(*t*)=*v* and *U*_*i*_(*t*)=*u*. Since the estimated *J*(*v*,*u*) only weakly depends on *u*, we visualize our results in figure 1 using two functions of one variable, *v*, namely

4.8 $$J_{1}(v)=J(v,0)\;\mathrm{and}\;J_{2}(v)=\int_{-\infty }^{\infty }J(v,u)\;p_{u}(u)\;du,$$

where *p*_*u*_(*u*) is the steady-state distribution of *U*_*i*_ estimated from the same long-time MD trajectory. As before, we use all three dimensions to calculate the averages *J*(*v*,*u*) and *p*_*u*_(*u*). Function *J*_1_(*v*) (which gives jerk at the most likely value of *U*_*i*_) is plotted using crosses and function *J*_2_(*v*), the average over *U*_*i*_ variable, is plotted using circles in figure 1. In order to compare all-atom MD simulations with the coarse-grained model (1.2)–(1.5), we calculate the corresponding jerk matrix *J*(*v*,*u*) for the coarse-grained model. We denote by *p*(*v*,*u*,*z*) the stationary distribution of the stochastic processes (1.3)–(1.5), i.e. *p*(*v*,*u*,*z*) *dv* *du* *dz* is the probability that *V*_*i*_(*t*)∈[*v*,*v*+*dv*), *U*_*i*_(*t*)∈[*u*,*u*+*du*) and *Z*_*i*_(*t*)∈[*z*,*z*+*dz*). Then the jerk matrix (4.6) of the coarse-grained model (1.2)–(1.5) is

$$J(v,u)=\int_{-\infty }^{\infty }\lim_{\tau \to 0}\frac{\left\langle U_{i}(t+\tau )-u\;|\;V_{i}(t)=v,U_{i}(t)=u,Z_{i}(t)=z\right\rangle }{\tau }\;p(v,u,z)\;dz.$$

Using (1.4), we rewrite it as

4.9 $$J(v,u)=\int_{-\infty }^{\infty }(-\eta_{1}v+z)\;p(v,u,z)\;dz.$$

The stationary distribution *p*(*v*,*u*,*z*) of (1.3)–(1.5) is Gaussian with mean (0,0,0) and stationary covariance matrix:

$$\frac{1}{2\eta_{1}\eta_{2}\eta_{3}}\left(\begin{array}{ccc} \eta_{4}^{2} & 0 & 0\\ 0 & \eta_{1}\eta_{4}^{2} & 0\\ 0 & 0 & \eta_{1}\eta_{3}\eta_{4}^{2} \end{array}\right).$$

Consequently, equation (4.9) implies

4.10 $$J(v,u)=-\eta_{1}v.$$

In figure 1, we plot (4.10) using the red solid line. The comparison with all-atom MD results (circles and squares) is excellent for all four ions considered in this paper. In particular, we have shown that the coarse-grained model (1.2)–(1.5) provides a good description of the rate of change of acceleration (jerk) at the MD timescale. We make use of this property in §6, where we use the same time step (Δ*t*=10^−3^ ps) for both the coarse-grained model (1.2)–(1.5) and all-atom MD simulations. The coarse-grained model (1.2)–(1.5) can also be coupled with BD description (1.1), which uses much larger timesteps, as we show in the next section.

## 5. From the coarse-grained model to Brownian dynamics

Let us consider the three-variable subsystem (1.3)–(1.5) of the coarse-grained model. Denoting **y**_*i*_=(*V*_*i*_,*U*_*i*_,*Z*_*i*_)^*T*^, where T stands for transpose, equations (1.3)–(1.5) can be written in vector notation as follows:

5.1 $$d{\text{y}}_{i}=B{\text{y}}_{i}\;dt+\text{b}\;dW_{i},$$

where matrix $B\in R^{3\times 3}$ and vector $\text{b}\in R^{3}$ are given as

5.2 $$B=\left(\begin{array}{ccc} 0 & 1 & 0\\ -\eta_{1} & 0 & 1\\ 0 & -\eta_{3} & -\eta_{2} \end{array}\right)\;\mathrm{and}\;\text{b}=\left(\begin{array}{c} 0\\ 0\\ \eta_{4} \end{array}\right).$$

Let us denote the eigenvalues and eigenvectors of *B* as *λ*_*j*_ and ***ν***_*j*_=(*ν*_1*j*_,*ν*_2*j*_,*ν*_3*j*_)^*T*^, *j*=1,2,3, respectively. The eigenvalues of *B* are the solutions of the characteristic polynomial

$$\lambda^{3}+\eta_{2}\;\lambda^{2}+(\eta_{1}+\eta_{3})\;\lambda +\eta_{1}\eta_{2}=0.$$

Since *η*_1_, *η*_2_ and *η*_3_ are positive parameters, we conclude that real parts of all three eigenvalues are negative and lie in interval (−*η*_2_,0). Using the values of *η*_*j*_, *j*=1,2,3, given in table 3, we present the values of eigenvalues *λ*_*j*_, *j*=1,2,3, in table 4. The eigenvalues *λ*_*j*_, *j*=1,2,3, are distinct. The general solution of the SDE system (5.1) can be written as follows [32]:

5.3 $${\text{y}}_{i}(t)=\Phi (t)\;\text{c}+\Phi (t)\int_{0}^{t}\Phi^{-1}(s)\;\text{b}\;dW_{i},$$

where $\text{c}\in R^{3}$ is a constant vector determined by initial conditions and matrix $\Phi (t)\in R^{3\times 3}$ is given as $\Phi (t)=(\exp(\lambda_{1}t)\nu_{1}\;|\;\exp(\lambda_{2}t)\nu_{2}\;|\;\exp(\lambda_{3}t)\nu_{3}),$ i.e. each column is a solution of the ODE system *d***y**_*i*_=*B***y**_*i*_ *dt*. Considering deterministic initial conditions, equation (5.3) implies that the process is Gaussian at any time *t*>0. Equations for means, variances and covariances then uniquely determine the distribution of **y**_*i*_(*t*) for *t*>0. Equations for means can be written in the vector form as *d*〈**y**_*i*_〉=*B*〈**y**_*i*_〉 *dt*. Equations for variances and covariances are given in §3 as equations (3.3)–(3.6), (3.9) and (3.16).

**Table 4.** Eigenvalues *λ*_*j*_, *j*=1,2,3, of matrix *B* defined by (5.2) and time shifts $t_{1}^{*}$ and $t_{2}^{*}.$ Symbol i denotes the imaginary unit.

ion	*λ*_1_ [ps^−1^]	*λ*_2_ [ps^−1^]	*λ*_3_ [ps^−1^]	$t_{1}^{*}$ [ps]	$t_{2}^{*}$ [ps]
K^+^	−127.0	−12.75+27.58 i	−12.75−27.58 i	3.08×10^−2^	−9.39×10^−3^
Na^+^	−140.1	−12.99+47.47 i	−12.99−47.47 i	6.15×10^−3^	−2.35×10^−2^
Ca^2+^	−163.1	−13.58+57.84 i	−13.58−57.84 i	1.47×10^−3^	−2.48×10^−2^
Cl^−^	−162.9	−13.41+30.25 i	−13.41−30.25 i	2.50×10^−2^	−1.13×10^−2^

There are two important conclusions of the above analysis. First of all, eigenvalues *λ*_*j*_, *j*=1,2,3, given in table 4 satisfy

λ1<Re λ2=Re λ3<0,

where Re denotes the real part of a complex number. There is a spectral gap between the first eigenvalue and the complex conjugate pair of eigenvalues. If we used this spectral gap, we could reduce the system to two evolution equations for times *t*≫1/|*λ*_1_|. However, there is no spectral gap to reduce the system to Langevin dynamics (4.1)–(4.2). In particular, we again confirm our conclusion that a coarse-grained approximation of ion behaviour is not given in terms of Langevin dynamics. Our second conclusion is that on a picosecond time scale, we can assume stationarity in (5.1) to get

5.4 $$dX_{i}=\frac{\eta_{4}}{\eta_{1}\eta_{2}}\;dW_{i},\;i=1,2,3.$$

Using (3.7), (3.15) and (3.17), we have

$$\frac{\eta_{4}}{\eta_{1}\eta_{2}}=\sqrt{2D}.$$

**Figure 2.**
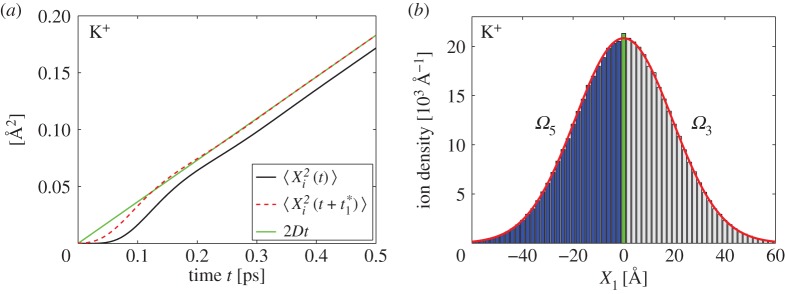
(*a*) Comparison of the coarse-grained model (1.2)–(1.5) and BD description (1.1) for K^+^ ion. The mean square displacement computed by solving 10 ODEs (3.3)–(3.6), (3.9), (3.11)–(3.14) and (3.16) with zero initial conditions (black solid line). The same curve shifted by the value of $t_{1}^{*}$ is plotted as a red dashed line. (*b*) Test of accuracy of the multiscale approach in *Ω*_3_∪*Ω*_4_∪*Ω*_5_ for K^+^ ion. Histogram obtained by simulating 10^6^ ions over time 10^3^ ps is compared with the analytical result (5.6) (red solid line). Grey bars show the ion density in *Ω*_3_, the green bar shows the ion density in *Ω*_4_ and blue bars show the ion density in *Ω*_5_. Parameters are given in tables 1 and 3.

Consequently, equation (5.4) is equivalent to BD description (1.1). The convergence of (1.2)–(1.5) to the BD model is illustrated in figure 2*a*. We solve the system of 10 ODEs for variances and covariances given as equations (3.3)–(3.6), (3.9), (3.11)–(3.14) and (3.16). We consider (deterministic) zero initial conditions, i.e. *X*_*i*_(0)=*V*_*i*_(0)=*U*_*i*_(0)=*Z*_*i*_(0)=0. All moments are then initially equal to zero. We plot the mean square displacement $\left\langle X_{i}^{2}\right\rangle$ as a function of time. We compare it with the mean square displacement of BD model (1.1) which is given as 2*Dt*. We observe that there is an approximately constant shift, denoted $t_{1}^{*},$ between both solutions for times *t*>0.2 ps. We illustrate this further by plotting $\left\langle X_{i}^{2}(t+t_{1}^{*})\right\rangle$ in figure 2*a*. The values of shift $t_{1}^{*}$ for different ions estimated by solving the ODEs for second moments with zero initial conditions are given in table 4.

Next, we show how the BD model (1.1) and the coarse-grained model (1.2)–(1.5) can be used in different parts of the computational domain. This coupling will form one component of multiscale methodology developed in §6. BD algorithms based on equation (1.1) have been implemented in a number of methods designed for spatio-temporal modelling of intracellular processes, including Smoldyn [33], MCell [34] and Green's-function reaction dynamics [35]. Smoldyn discretizes (1.1) using a fixed BD timestep Δ*T*, i.e. it computes the time evolution of the position **X**≡**X**(*t*) of each molecule by

5.5 $$X_{i}(t+\Delta T)=X_{i}(t)+\sqrt{2D\Delta T}\;\xi_{i},\;i=1,2,3,$$

where (*ξ*_1_,*ξ*_2_,*ξ*_3_) is a vector of normally distributed random numbers with zero mean and unit variance. We use discretization (5.5) of BD model (1.1) in this paper. BD time step Δ*T* has to be chosen much larger than the MD timestep Δ*t*. We use Δ*T*=0.5 ps, but any larger timestep would also work well. We could also use a variable timestep, as implemented in the Green's-function reaction dynamics [35].

In §6, we consider all-atom MD simulations in domain $\Omega \subset R^{3}$. Our main goal is to design a multiscale approach which can compute spatio-temporal statistics with the MD-level of detail in relatively small subdomain *Ω*_1_⊂*Ω* by using BD model (5.5) in the most of the rest of the computational domain. This is achieved by decomposing domain *Ω* into five subdomains *Ω*_*j*_, *j*=1,2,3,4,5 (see equation (6.1) and discussion in §6). We use MD in *Ω*_1_, the coarse-grained model (1.2)–(1.5) in *Ω*_3_ and the BD model (5.5) in *Ω*_5_. The remaining two subdomains, *Ω*_2_ and *Ω*_4_, are two overlap (hand-shaking) regions where two different simulation approaches can be used at the same time [12,36]. In the rest of this section, we focus on simulations in region *Ω*_3_∪*Ω*_4_∪*Ω*_5_ which concerns coupling the coarse-grained model (1.2)–(1.5) with the BD model (5.5). We use the coarse-grained model in *Ω*_3_∪*Ω*_4_ and the BD model (5.5) in *Ω*_4_∪*Ω*_5_. In particular, we use both models in the overlap region *Ω*_4_. Each particle which is initially in *Ω*_3_ is simulated according to (1.2)–(1.5) (discretized using timestep Δ*t*) until it enters *Ω*_5_. Then we use (5.5) to evolve the position of a particle (over BD timesteps of length Δ*T*) until it again enters *Ω*_3_ when we switch the description back from the BD model to the coarse-grained model. In order to do this, we have to initialize variables *V*_*i*_, *U*_*i*_ and *Z*_*i*_, *i*=1,2,3. We use deterministic initial conditions, *V*_*i*_(0)=*U*_*i*_(0)=*Z*_*i*_(0)=0, discussed above.

In figure 2*b*, we present an illustrative simulation where $\Omega_{3}\cup \Omega_{4}\cup \Omega_{5}=R^{3}$ for simplicity. We use $\Omega_{3}=[h,\infty )\times R^{2},$ $\Omega_{4}=(-h,h)\times R^{2}$ and $\Omega_{5}=(-\infty ,-h]\times R^{2},$ where *h*=1 Å. We report averages over 10^6^ simulations of ions, half of them are initiated at **X**(0)=(*h*,0,0), i.e. they initially follow the coarse-grained model (1.2)–(1.5) with zero initial condition for other variables (*V*_*i*_(0)=*U*_*i*_(0)=*Z*_*i*_(0)=0). The second half of ions are initiated at **X**(0)=(−*h*,0,0), i.e. they initially follow BD description (5.5). We plot the (marginal) distribution of ions along the first coordinate (*X*_1_) at time 10^3^ ps in figure 2*b*. The computed histogram is plotted using bins of length 2 Å, i.e. the overlap region *Ω*_4_ is equal to one bin (visualized as a green bar). Grey (respectively, blue) bars show the density of ions in *Ω*_3_ (respectively, *Ω*_5_). We compare our results with the analytical distribution computed for BD description (1.1) at time *t*=10^3^ ps given by

5.6 $$\varrho (x_{1})=\frac{10^{6}}{4\sqrt{\pi Dt}}\left(\exp\left[-\frac{{\left(x_{1}-h\right)}^{2}}{4Dt}\right]+\exp\left[-\frac{{\left(x_{1}+h\right)}^{2}}{4Dt}\right]\right).$$

The computed histogram compares well with (5.6), although we can observe a small error: the green bar is slightly taller than the corresponding value of (5.6). If we wanted to further improve the accuracy, we could take into account that there is time shift $t_{1}^{*},$ discussed above, introduced to the multiscale approach by using the deterministic initial conditions, *V*_*i*_(0)=*U*_*i*_(0)=*Z*_*i*_(0)=0, for ions entering domain *Ω*_3_. Another possibility is to sample the initial condition for *V*_*i*_, *U*_*i*_ and *Z*_*i*_ from a suitable distribution. If we use the stationary distribution of subsystem (1.3)–(1.5), then $\left\langle V_{i}^{2}\right\rangle$ does not evolve and is equal to

$$\langle V_{i}^{2}\rangle =\frac{\eta_{4}^{2}}{2\eta_{1}\eta_{2}\eta_{3}}.$$

Substituting this constant for $\left\langle V_{i}^{2}\right\rangle$ into (3.12), the system of 10 ODEs for second moments of (1.2)–(1.5) simplifies to four ODEs (3.11)–(3.14). Solving system (3.11)–(3.14) with zero initial conditions (assuming *X*_*i*_(0)=0), we can again compute the mean square displacement. As in figure 2*a*, it can be shifted in time to better match with the BD result, 2*Dt*. We denote this time shift as $t_{2}^{*}$. Its values are given in table 4. We observe that $t_{2}^{*}$ is negative and $t_{1}^{*}$ is positive for all four ions considered in table 4. Both time shifts $t_{1}^{*}$ and $t_{2}^{*}$ (together with optimizing size *h* of the overlap region) could be used to further improve the accuracy of multiscale simulations in *Ω*_3_∪*Ω*_4_∪*Ω*_5_ [12]. However, our main goal is to introduce a multiscale approach which can use all-atom MD simulations in *Ω*_1_. Since MD simulations are computationally intensive, we will only consider 100 realizations of the multiscale method in §6. In particular, the Monte Carlo error will be larger than the error observed in figure 2*b*. Thus, we can use the above approach in *Ω*_3_∪*Ω*_4_∪*Ω*_5_ without introducing observable errors in the multiscale method developed in the next section.

## 6. Coupling all-atom MD and BD

**Figure 3.**
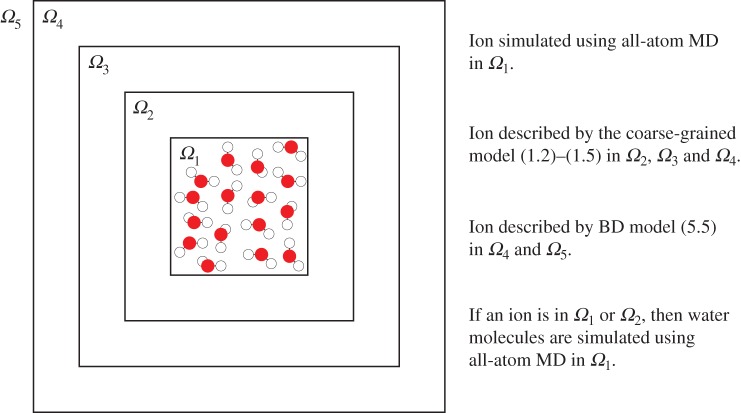
Schematic of multiscale set-up. Note that the schematic is drawn in two spatial dimensions to enable better visualization, but all models are formulated and simulated in three spatial dimensions. (Online version in colour.)

Let us consider all-atom MD in domain $\Omega \subset R^{3}$ which is so large that direct MD simulations would be too computationally expensive. Let us assume that a modeller only needs to consider the MD-level of detail in a relatively small subdomain *Ω*_1_⊂*Ω*, while, in the rest of the computational domain, ions are transported by diffusion and BD description (1.1) is applicable. For example, domain *Ω*_1_ could include binding sites for ions or (parts of) ion channels. In this paper, we do not focus on a specific application. Our goal is to show that the coarse-grained model (1.2)–(1.5) is an intermediate model between all-atom MD and BD which enables the use of both methods during the same dynamic simulation. To achieve this, we decompose domain *Ω* into five subdomains, denoted as *Ω*_*j*_, *j*=1,2,3,4,5 (as it is schematically illustrated in figure 3). These sets are considered pairwise disjoint (i.e. *Ω*_*i*_∩*Ω*_*j*_=∅ for *i*≠*j*) and

6.1 $$\Omega =\Omega_{1}\cup \Omega_{2}\cup \Omega_{3}\cup \Omega_{4}\cup \Omega_{5}.$$

In our illustrative simulations, we consider the behaviour of one ion. If the ion is in *Ω*_1_, then we use all-atom MD simulations as described in §2. In particular, the force between the ion and a water molecule is obtained by differentiating potential (2.1), provided that the distance between the ion and the water molecule is less than the cut-off distance (*L*/2). Let us denote the force exerted by the ion on the water molecule by **F**_iw_(*r*_i0_,*r*_i1_,*r*_i2_), where *r*_i0_ (respectively, *r*_i1_ and *r*_i2_) is the distance between the ion and the oxygen site (respectively, the first and second hydrogen sites) on the water molecule. We use periodic boundary conditions for water molecules in *Ω*_1_.

Whenever the ion leaves *Ω*_1_, it enters *Ω*_2_ where we simulate its behaviour using the coarse-grained model (1.2)–(1.5). We still simulate water molecules in *Ω*_1_ and we allow them to experience additional forces exerted by the ion which is present in *Ω*_2_. These forces have the same functional form, **F**_iw_, as in MD, but they have modified arguments as follows:

6.2 $${\text{F}}_{\mathrm{iw}}(r_{i0}+\omega \;\mathrm{dist}(\text{X},\Omega_{1}),r_{i1}+\omega \;\mathrm{dist}(\text{X},\Omega_{1}),r_{i2}+\omega \;\mathrm{dist}(\text{X},\Omega_{1})),$$

where *ω*≥0 is a parameter and dist(**X**,*Ω*_1_) is the (closest) distance between the ion at position **X** and subdomain *Ω*_1_. If the ion is in region *Ω*_3_∪*Ω*_4_∪*Ω*_5_, then water molecules in *Ω*_1_ are no longer simulated. We use the coarse-grained model (1.2)–(1.5) to simulate the ion behaviour in *Ω*_3_ and the BD model (5.5) in *Ω*_5_. Overlap region *Ω*_4_ is used to couple these simulation methods as explained in §5.

In §5, we have already presented illustrative simulations to validate the multiscale modelling strategy chosen in region *Ω*_3_∪*Ω*_4_∪*Ω*_5_. Next, we focus on testing and explaining the multiscale approach chosen to couple region *Ω*_1_ with *Ω*_2_. The key idea is given by force term (6.2) which is used for MD simulations of water molecules in *Ω*_1_ when an ion is in *Ω*_2_. This force term has two important properties:

(i) If an ion is on the boundary of *Ω*_1_, i.e. **X**∈∂*Ω*_1_, then dist(**X**,*Ω*_1_)=0 and force (6.2) is equal to force term **F**_iw_(*r*_i0_,*r*_i1_,*r*_i2_) used in *Ω*_1_.
(ii) If *ω* dist(**X**,*Ω*_1_)≥*L*/2, then force (6.2) is equal to zero.

Property (i) implies that formula (6.2) continuously extends the force term used in MD. In particular, water molecules do not experience abrupt changes of forces when the ion crosses boundary ∂*Ω*_1_. Property (ii) is a consequence of the cut-off distance used (together with the reaction field correction [1]) to treat long-range interactions. In our illustrative simulations, we use

6.3 $$\Omega_{1}={\left[-\frac{L}{2},\frac{L}{2}\right]}^{3}\;\mathrm{and}\;\Omega_{2}={\left[-\frac{L}{2}-\frac{L}{2\omega },\frac{L}{2}+\frac{L}{2\omega }\right]}^{3}\setminus \;\Omega_{1}.$$

Property (ii) implies that extra force (6.2) is equal to zero on boundary ∂*Ω*_2_∖∂*Ω*_1_ which is the boundary between regions *Ω*_2_ and *Ω*_3_. This is consistent with the assumption that ions in region *Ω*_3_∪*Ω*_4_∪*Ω*_5_ do not interact with water molecules in region *Ω*_1_.

If an ion is in *Ω*_1_, we use all-atom MD as formulated in §2. Periodic boundary conditions are implemented in MD simulations. Water molecules are subject to forces exerted not only by the ion at its real position **X** in *Ω*_1_, but also by its copies at periodic locations **X**+(*iL*,*jL*,*kL*), where i,j,k∈Z. When the ion moves to *Ω*_2_, one of its copies is in *Ω*_1_. Force term (6.2) is designed in such a way that the strength of interaction decreases (for every copy of the ion) with the distance, dist(**X**,*Ω*_1_), between the real position of the ion and *Ω*_1_. In particular, force term (6.2) ensures that there are continuous changes of all forces when the ion moves between regions *Ω*_1_, *Ω*_2_ and *Ω*_3_.

**Figure 4.**
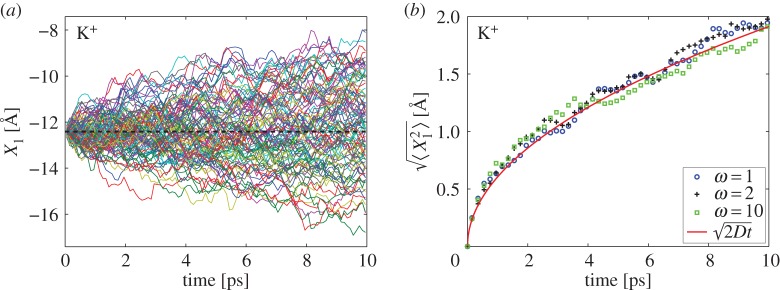
(*a*) One hundred realizations of a multiscale simulation of K^+^ ion initiated at (−*L*/2,0,0). We plot *X*_1_ coordinate as a function of time. Ion is described by all-atom MD for *X*_1_≥−*L*/2 and by the coarse-grained model (1.2)–(1.5) for *X*_1_<−*L*/2. The boundary between *Ω*_1_ and *Ω*_2_ is visualized using the black dashed line. We use *ω*=1 in (6.2). (*b*) The mean square displacement in the first coordinate of K^+^ ion simulated in *Ω*_1_∪*Ω*_2_ and computed as the average of 100 realizations for *ω*=1 (blue circles), *ω*=2 (black crosses) and *ω*=10 (green squares).

In figure 4, we present results of simulations of K^+^ ion in region *Ω*_1_∪*Ω*_2_. We consider 100 realizations of a multiscale simulation with one ion. Its initial position is **X**(0)=(−*L*/2,0,0) which lies on boundary ∂*Ω*_1_. We simulate each realization for time 10 ps which is short enough that all trajectories stay inside the ball of radius *L*/2 centred at **X**(0). Then *X*_1_-coordinate of the trajectory determines whether the ion is in *Ω*_1_ or *Ω*_2_. If *X*_1_(*t*)≥−*L*/2, then the ion is in *Ω*_1_ and it is simulated using all-atom MD. If *X*_2_(*t*)<−*L*/2, then the ion is in *Ω*_2_ and evolves according to the coarse-grained model (1.2)–(1.5). In figure 4*a*, we use (6.2) with *ω*=1 and plot *X*_1_ coordinates of all 100 realizations. We observe that the computed trajectories spread on both sides of boundary ∂*Ω*_1_ (dashed line) without any significant bias. The mean square displacement is presented in figure 4*b* for three different values of *ω*. The results compare well with (2*Dt*)^1/2^ which is the mean square displacement of one coordinate of the diffusion process.

**Figure 5.**
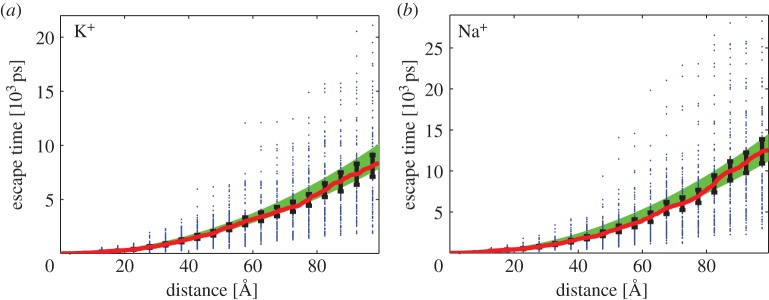
Escape time T(r) to reach distance *r* from the origin computed by the multiscale method. We consider (*a*) K^+^ ion; and (*b*) Na^+^ ion. We plot escape times for individual realizations (blue points), the mean escape time estimated from 100 realizations (red solid line) and the theoretical 95% confidence interval (6.4) (green area). We use *ω*=10 in (6.2).

We conclude with illustrative simulations which are coupling all-atom MD with BD. We use domain $\Omega \in R^{3}$ decomposed into five regions as in equation (6.1), where *Ω*_1_ and *Ω*_2_ are given by (6.3), and

$$\begin{array}{rl} \Omega_{3} & ={\left[-\frac{L}{2}-\frac{L}{2\omega }-h_{1},\frac{L}{2}+\frac{L}{2\omega }+h_{1}\right]}^{3}\setminus \;(\Omega_{1}\cup \Omega_{2}),\\ \Omega_{4} & ={\left[-\frac{L}{2}-\frac{L}{2\omega }-h_{1}-h_{2},\frac{L}{2}+\frac{L}{2\omega }+h_{1}+h_{2}\right]}^{3}\setminus \;(\Omega_{1}\cup \Omega_{2}\cup \Omega_{3}),\\ \Omega_{5} & =R^{3}\setminus (\Omega_{1}\cup \Omega_{2}\cup \Omega_{3}\cup \Omega_{4}), \end{array}$$

where *ω*=10, *h*_1_=*L*/20 and *h*_2_=*L*/10. Then the BD domain is $\Omega_{5}=R^{3}\setminus {\left[-7L/10,7L/10\right]}^{3}$. We place an ion at the origin (centre of MD domain *Ω*_1_), i.e. **X**(0)=(0,0,0), and we simulate each trajectory until it reaches the distance 4*L*=99.32 Åfrom the origin. Let T(r) be the time when a trajectory first reaches distance *r* from the origin. In figure 5, we plot escape time T(r) as a function of distance *r*. We plot the value of T(r) for each realization as a blue point. The largest computed escape times (for *r*=4*L*) are 38 506 ps for K^+^ and 47 212 ps for Na^+^. They are outside the range of panels in figure 5, but the majority of data points are included in this figure. We also plot average ⟨T(r)⟩ (red solid line) together with 95% confidence intervals. They are compared with theoretical results obtained for the BD model (1.1). The escape time distribution for the BD model (1.1) has mean equal to $\left\langle T(r)\rangle =r^{2}/(6D\right)$ and standard deviation $r^{2}/(3\sqrt{10}\;D).$ The corresponding theoretical 95% confidence interval (for 100 samples) is

6.4 $$\left(\frac{r^{2}}{6D}-1.96\frac{r^{2}}{30D},\;\frac{r^{2}}{6D}+1.96\frac{r^{2}}{30D}\right).$$

This interval is visualized as the green area in figure 5. We note that it would be relatively straightforward to continue the presented multiscale computation and simulate ion diffusion in domains covering the whole cell. The most computationally intensive part is all-atom MD simulation in *Ω*_1_∪*Ω*_2_. However, once the ion enters *Ω*_5_, we can compute its trajectory very efficiently. We could further increase the BD timestep in parts of *Ω*_5_ which are far away from *Ω*_4_, or we could use event-based algorithms, like Green's-function reaction dynamics [35] or First-passage kinetic Monte Carlo method [37], to compute the ion trajectory in region *Ω*_5_.

## 7. A hierarchy of stochastic coarse-grained models

In §6, the coarse-grained model (1.2)–(1.5) has been used in intermediate regions (denoted *Ω*_2_, *Ω*_3_ and *Ω*_4_ in figure 3) to couple all-atom MD and BD. It is a simple model which has all the necessary properties for this task. The developed multiscale algorithm enables the use of MD together with modern spatio-temporal simulation algorithms for intracellular processes. The coarse-grained model (1.2)–(1.5) (parametrized by four constants *η*_1_, *η*_2_, *η*_3_ and *η*_4_) provides a better description of ion dynamics than BD (parametrized by one constant *D*). However, it does not capture all the details of MD. In this section, we introduce a hierarchy of coarse-grained models which generalize (1.2)–(1.5) and include an increasing number of parameters. We illustrate that these models can be used to fit additional characteristics of all-atom MD simulations.

There has been a lot of approaches in the literature to develop coarse-grained models at equilibrium by constructing suitable coarse-grained potential energy functions with fewer degrees of freedom [38–40]. However, the same coarse-grained potential energy function can correspond to many different dynamic coarse-grained models. In particular, the conservative Hamiltonian dynamics of coarse-grained variables on a coarse-grained potential surface is usually a poor approximation of the real dynamics of all-atom MD models [41,42]. In recent work, Davtyan *et al.* [13] use fictitious particles with harmonic interactions with coarse-grained degrees of freedom (i.e. they add quadratic terms to the potential function of the system) to improve the fit between an MD model and the dynamics on a coarse-grained potential surface. Each fictitious particle is also subject to a friction force and noise. We will apply the fictitious particle approach to develop a hierarchy of coarse-grained models which generalize (1.2)–(1.5).

Let us consider *N* fictitious particles interacting with an ion at position $X\equiv (X_{1},X_{2},X_{3})$ with velocity $V\equiv (V_{1},V_{2},V_{3})$. Let ${\tilde{X}}_{j}\equiv ({\tilde{X}}_{j,1},{\tilde{X}}_{j,2},{\tilde{X}}_{j,3})$ be the position of the *j*th fictitious particle, j=1,2,…,N. Let ${\tilde{V}}_{j}\equiv ({\tilde{V}}_{j,1},{\tilde{V}}_{j,2},{\tilde{V}}_{j,3})$ be its velocity. Then the fictitious particle model [13] of an ion can be written as the following set of 6(*N*+1) equations (for *i*=1,2,3 and j=1,2,…,N)

7.1 $$\begin{array}{rl} dX_{i} & =V_{i}\;dt, \end{array}$$

7.2 $$\begin{array}{rl} dV_{i} & =\sum_{j=1}^{N}\alpha_{j,1}({\tilde{X}}_{j,i}-X_{i})\;dt, \end{array}$$

7.3 $$\begin{array}{rl} d{\tilde{X}}_{j,i} & ={\tilde{V}}_{j,i}\;dt \end{array}$$

7.4 $$\begin{array}{rl} \text{and}\;\;d{\tilde{V}}_{j,i} & =-(\alpha_{j,2}{\tilde{V}}_{j,i}+\alpha_{j,3}({\tilde{X}}_{j,i}-X_{i}))\;dt+\alpha_{j,4}\;dW_{j,i}, \end{array}$$

where white noise vectors d**W**_*j*_≡(d*W*_*j*,1_,d*W*_*j*,2_,d*W*_*j*,3_) are mutually independent. Each fictitious particle can be characterized by four constants: three of them correspond to three force terms on the right-hand side of (7.4) and the fourth one is the fictitious particle mass. In order to write the fictitious particle model in a similar way as other models in this paper, we adsorbed the masses of the ion and fictitious particles into the constants on the right-hand sides of equations (7.2) and (7.4). In particular, the *j*th fictitious particle is characterized by four positive constants *α*_*j*,1_, *α*_*j*,2_, *α*_*j*,3_ and *α*_*j*,4_. To connect the hierarchy of the stochastic fictitious particle models (7.1)–(7.4) with the coarse-grained model (1.2)–(1.5), we introduce new variables ${\tilde{U}}_{j,i}=\alpha_{j,1}({\tilde{X}}_{j,i}-X_{i})$ and ${\tilde{Z}}_{j,i}=\alpha_{j,1}{\tilde{V}}_{j,i}$. Then equations (7.2)–(7.4) read as follows:

7.5 $$\begin{array}{rl} dV_{i} & =\sum_{j=1}^{N}{\tilde{U}}_{j,i}\;dt, \end{array}$$

7.6 $$\begin{array}{rl} d{\tilde{U}}_{j,i} & =({\tilde{Z}}_{j,i}-\alpha_{j,1}V_{i})\;dt \end{array}$$

7.7 $$\begin{array}{rl} \text{and}\;\;d{\tilde{Z}}_{j,i} & =-(\alpha_{j,2}{\tilde{Z}}_{j,i}+\alpha_{j,3}{\tilde{U}}_{j,i})\;dt+\alpha_{j,1}\alpha_{j,4}\;dW_{j,i}. \end{array}$$

Using *N*=1 and denoting $U_{i}={\tilde{U}}_{1,i}$ and $Z_{i}={\tilde{Z}}_{1,i},$ we obtain the coarse-grained model (1.2)–(1.5) where *η*_1_=*α*_1,1_, *η*_2_=*α*_1,2_, *η*_3_=*α*_1,3_ and *η*_4_=*α*_1,1_*α*_1,4_. Thus, the coarse-grained model (1.2)–(1.5) is equivalent to the fictitious particle model (7.1)–(7.4) for *N*=1. Moreover, stochastic coarse-grained models in the hierarchy (7.1)–(7.4) provide generalizations of the coarse-grained model (1.2)–(1.5) for *N*≥2 and can be used to fit additional details of MD simulations.

One commonly used MD characteristic is the velocity autocorrelation function

7.8 $$C(t)=\langle V_{i}(t)V_{i}(0)\rangle .$$

**Figure 6.**
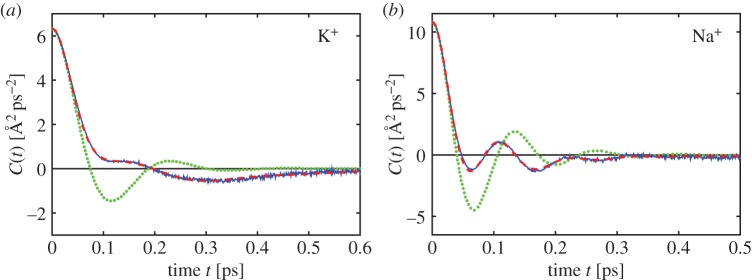
(*a*) Velocity autocorrelation function (7.8) calculated by the all-atom MD model of K^+^ ion (blue solid line), by the coarse-grained model (1.2)–(1.5) (green dotted line) and by the fictitious particle model (7.1)–(7.4) for *N*=3 (red dashed line), where the parameters of (7.1)–(7.4) are *α*_1,1_=5.64×10^2^ ps^−2^, *α*_1,2_=73.8 ps^−1^, *α*_1,3_=3.42×10^3^ ps^−2^, *α*_1,4_=80.9 Å ps^−3/2^, *α*_2,1_=1.26× 10^2^ ps^−2^, *α*_2,2_=21.3 ps^−1^, *α*_2,3_=7.27×10^2^ ps^−2^, *α*_2,4_= 3.43×10^−1^ Å ps^−3/2^, *α*_3,1_=72.1 ps^−2^, *α*_3,2_=2.02×10^2^ ps^−1^, *α*_3,3_=3.26×10^−1^ ps^−2^ and *α*_3,4_=1.22× 10^2^ Å ps^−3/2^. The parameters of the coarse-grained model (1.2)–(1.5) are given in table 3. (*b*) The same computations for Na^+^ ion, where the parameters of the fictitious particle model (7.1)–(7.4) are *α*_1,1_=3.05×10^3^ ps^−2^, *α*_1,2_=3.08×10^2^ ps^−1^, *α*_1,3_=8.99× 10^3^ ps^−2^, *α*_1,4_=98.7 Å ps^−3/2^, *α*_2,1_= 96.5 ps^−2^, *α*_2,2_=2.48×10^2^ ps^−1^, *α*_2,3_=4.62×10^2^ ps^−2^, *α*_2,4_=15.8 Å ps^−3/2^, *α*_3,1_=23.5 ps^−2^, *α*_3,2_=25.5 ps^−1^, *α*_3,3_=3.82×10^2^ ps^−2^ and *α*_3,4_=1.18×10^3^ Å ps^−3/2^.

In figure 6, we plot the velocity autocorrelation function estimated from MD simulations of K^+^ and Na^+^ ions. Using (5.3), we can find an analytical expression for the velocity autocorrelation function of the coarse-grained model (1.2)–(1.5) as follows:

7.9 $$C(t)=(1,0,0)\;\Phi (t)\;\Phi^{-1}(0)\left(\begin{array}{c} \left\langle V_{i}^{2}\right\rangle \\ 0\\ 0 \end{array}\right),$$

where matrix $\Phi (t)\in R^{3\times 3}$ is given as $\Phi (t)=(\exp(\lambda_{1}t)\nu_{1}\;|\;\exp(\lambda_{2}t)\nu_{2}\;|\;\exp(\lambda_{3}t)\nu_{3}),$ where *λ*_*j*_, *j*=1,2,3, are eigenvalues of matrix *B* given by (5.2) and ***ν***_*j*_=(*ν*_1*j*_,*ν*_2*j*_,*ν*_3*j*_)^*T*^ are the corresponding eigenvectors. In figure 6, we plot velocity autocorrelation functions (7.9) as green dotted lines for K^+^ and Na^+^ ions.

Using *t*=0 in (7.9), we obtain $C(0)=\langle V_{i}^{2}\rangle$. Since we have parametrized the coarse-grained model (1.2)–(1.5) to give the same value of $\left\langle V_{i}^{2}\right\rangle$ as obtained by the corresponding MD model, the velocity autocorrelation function of the coarse-grained model agrees with MD simulations for *t*=0. In figure 6, we observe that the coarse-grained model approximates *C*(*t*) well for *t* between 0 and 30 fs. It is also a good approximation for large values of *t*, because *C*(*t*)→0 as t→∞, but we can clearly see a difference for intermediate values of *t*. Since we have parametrized the coarse-grained model (1.2)–(1.5) by fitting the diffusion constant *D*, the coarse-grained model also approximates well the integral of *C*(*t*), because of the Green–Kubo formula

$$D=\int_{0}^{\infty }\langle V_{i}(t)V_{i}(0)\rangle \;dt=\int_{0}^{\infty }C(t)\;dt.$$

If a modeller wants to improve the approximation of *C*(*t*) for intermediate values of *t*, the fictitious particle model (7.1)–(7.4) can be used for *N*>1 as a generalization of the coarse-grained model (1.2)–(1.5). We will illustrate this by using *N*=3. Equations (7.5)–(7.7) can then be rewritten in vector notation as follows (cf. (5.1)):

7.10 $$d{\tilde{\text{y}}}_{i}=\tilde{B}\;{\tilde{\text{y}}}_{i}\;dt+{\tilde{b}}^{T}\;d\tilde{{\text{W}}_{i}},$$

where ${\tilde{\text{y}}}_{i}={\left(V_{i},{\tilde{U}}_{1,i},{\tilde{Z}}_{1,i},{\tilde{U}}_{2,i},{\tilde{Z}}_{2,i},{\tilde{U}}_{3,i},{\tilde{Z}}_{3,i}\right)}^{T}$,

$$\tilde{b}=\left(\begin{array}{ccccccc} 0 & 0 & \alpha_{1,1}\alpha_{1,4} & 0 & 0 & 0 & 0\\ 0 & 0 & 0 & 0 & \alpha_{2,1}\alpha_{2,4} & 0 & 0\\ 0 & 0 & 0 & 0 & 0 & 0 & \alpha_{3,1}\alpha_{3,4} \end{array}\right),\;d\tilde{{\text{W}}_{i}}=\left(\begin{array}{c} dW_{1,i}\\ dW_{2,i}\\ dW_{3,i} \end{array}\right),$$

and matrix $\tilde{B}\in R^{7\times 7}$ is given as (cf. (5.2))

$$\tilde{B}=\left(\begin{array}{ccccccc} 0 & 1 & 0 & 1 & 0 & 1 & 0\\ -\alpha_{1,1} & 0 & 1 & 0 & 0 & 0 & 0\\ 0 & -\alpha_{1,3} & -\alpha_{1,2} & 0 & 0 & 0 & 0\\ -\alpha_{2,1} & 0 & 0 & 0 & 1 & 0 & 0\\ 0 & 0 & 0 & -\alpha_{2,3} & -\alpha_{2,2} & 0 & 0\\ -\alpha_{3,1} & 0 & 0 & 0 & 0 & 0 & 1\\ 0 & 0 & 0 & 0 & 0 & -\alpha_{3,3} & -\alpha_{3,2} \end{array}\right).$$

Let us denote the eigenvalues and eigenvectors of $\tilde{B}$ as ${\tilde{\lambda }}_{j}$ and ${\tilde{\nu }}_{j},$ j=1,2,…,7, respectively. In what follows, we will assume that coefficients *α*_*j*,*k*_ are chosen so that the eigenvalues of $\tilde{B}$ are distinct and have negative real parts. Then the velocity autocorrelation function of the fictitious particle model (7.10) can be computed as follows (cf. (7.9))

7.11 $$C(t)=(1,0,0,0,0,0,0)\;\tilde{\Phi }(t)\;{\tilde{\Phi }}^{-1}(0)\;\langle V_{i}{\tilde{\text{y}}}_{i}\rangle ,$$

where matrix $\tilde{\Phi }(t)\in R^{7\times 7}$ is given as $\tilde{\Phi }(t)=(\exp({\tilde{\lambda }}_{1}t){\tilde{\nu }}_{1}\;|\;\exp({\tilde{\lambda }}_{2}t){\tilde{\nu }}_{2}\;|\;\cdots \;|\;\exp({\tilde{\lambda }}_{7}t){\tilde{\nu }}_{7}),$ i.e. each column is a solution of the ODE system $d{\tilde{\text{y}}}_{i}=\tilde{B}{\tilde{\text{y}}}_{i}\;dt$. Vector $\left\langle V_{i}{\tilde{\text{y}}}_{i}\right\rangle$ is the first column of the equilibrium covariance matrix $\left\langle {\tilde{\text{y}}}_{i}{\tilde{\text{y}}}_{i}^{T}\right\rangle$ which can be obtained as the solution of the linear system

7.12 $$\tilde{B}\langle {\tilde{\text{y}}}_{i}{\tilde{\text{y}}}_{i}^{T}\rangle +\langle {\tilde{\text{y}}}_{i}{\tilde{\text{y}}}_{i}^{T}\rangle {\tilde{B}}^{T}=-{\tilde{b}}^{T}\tilde{b}.$$

Since we want the fictitious particle model (7.1)–(7.4) to be a generalization of the coarse-grained models (1.2)–(1.5), we select its parameters so that the model (7.1)–(7.4) has the same diffusion constant *D* and moments $\left\langle V_{i}^{2}\right\rangle$ and $\left\langle U_{i}^{2}\right\rangle$ as calculated by all-atom MD simulations in table 2. This yields three conditions between 12 parameters *α*_*j*,*k*_, *j*=1,2,3, *k*=1,2,3,4, of the fictitious particle model (7.1)–(7.4). In particular, we have a lot of freedom to choose the 12 model parameters. In figure 6, we plot the velocity autocorrelation function (7.11) for specific choices of parameters *α*_*j*,*k*_, illustrating that we can select these parameters to approximate velocity autocorrelation functions obtained by all-atom MD. To obtain these results, we use a simple acceptance–rejection algorithm based on equations (7.11)–(7.12). We start with an initial guess of 12 parameters *α*_*j*,*k*_ and calculate the *L*^1^-error between the velocity autocorrelation function (7.11) and the MD result (blue solid line in figure 6). Then we perturb the parameters *α*_*j*,*k*_ in a way that the resulting model still has the same *D*, $\left\langle V_{i}^{2}\right\rangle$ and $\left\langle U_{i}^{2}\right\rangle$ and we use the new set of parameters *α*_*j*,*k*_ to recalculate the *L*^1^-error between the velocity autocorrelation function (7.11) and the MD result. If the error decreases, then we accept the new set of parameters. We iterate these steps until the desired level of error.

In figure 6, we observe that the velocity autocorrelation function computed by (7.11) for *N*=3 (red dashed line) approximates well the MD result. In a similar way, we could fit other autocorrelation functions (if required) by coarse-grained models in the hierarchy (7.1)–(7.4) for sufficiently large *N*. However, as we have seen in §6, coupling all-atom MD and BD can be achieved by the coarse-grained model (1.2)–(1.5), i.e. by using *N*=1.

## 8. Discussion

In this paper, we have introduced and studied the coarse-grained model (1.2)–(1.5) of an ion in aqueous solution. We have parametrized this model using all-atom MD simulations for four ions (K^+^, Na^+^, Ca^2+^ and Cl^−^) and showed that this model provides an intermediate description between all-atom MD and BD simulations. It can be used both with MD timestep Δ*t* (to couple it with all-atom MD simulations) and BD time step Δ*T* (to couple it with BD description (1.1)). In particular, the coarse-grained model enables multiscale simulations which use all-atom MD and BD in different parts of the computational domain.

In §6, we have illustrated this multiscale methodology using a first passage type problem where we have reported the time taken by an ion to reach a specific distance. Possible applications of this multiscale methodogy include problems where a modeller considers all-atom MD in several different parts of the cell (e.g. close to binding sites or ion channels) and wants to use efficient BD simulations to transport ions by diffusion between regions where MD is used. The proposed approach thus enables the inclusion of MD-level of detail in computational domains which are much larger than would be possible to study by direct MD simulations.

Although the illustrative simulations in §6 are reported over distances of the order of 10^2^ Å, this is not a restriction of the method. Most of the computational time is spent by considering all-atom MD in *Ω*_1_∪*Ω*_2_. BD uses a much larger timestep which enables us to further extend the BD region *Ω*_5_ (and consequently, the original domain *Ω*). Moreover, if we are far away from the MD domain *Ω*_1_, we can further increase the efficiency of BD simulations by using different BD timesteps in different parts of the BD subdomain *Ω*_5_ [12], or by using event-based BD algorithms [35,37]. The computational intensity of BD simulations can be further decreased by using multiscale methods which efficiently and accurately combine BD models with lattice-based (compartment-based) models [43,44]. Such a strategy has been previously used for modelling intracellular calcium dynamics [3,7] or actin dynamics in filopodia [45], and enables us to extend both temporal and spatial extents of the simulation.

In §7, we have presented a systematic procedure for including additional auxiliary variables and parameters to the coarse-grained model (1.2)–(1.5). We have illustrated that the generalized models can be used to fit additional details of all-atom MD simulations. The generalization of the coarse-grained model (1.2)–(1.5) is based on the fictitious particle approach [13] which introduces fictitious particles with harmonic interactions with coarse-grained degrees of freedom. Such special heat baths have been previously studied in the context of the generalized Langevin equation [14–17]. In §7, we have shown that an appropriately formulated fictitious particle model which uses one fictitious particle per ion [13] has the same dynamics as the coarse-grained model (1.2)–(1.5).

In the literature, MD methods have been used to estimate parameters of BD simulations of ions [46]. There has also been a lot of progress in systematic coarse-graining of MD simulations [40,47]. The approach presented in this paper not only uses all-atom MD simulations to estimate parameters of a coarser description, but it also designs a multiscale approach where both methods are used during the same simulation. Methods which adaptively change the resolution of MD on demand have been previously reported in [48,49]. They include algorithms which couple all-atom MD with coarse-grained MD. The coarse-grained model studied in this work does not include any water molecules and has different application areas. One of them is modelling of calcium induced calcium release through IP_3_R channels [3] which is discussed as a motivating example in §1. MD simulations in this paper use the three-site SPC/E model of water. An open question is to extend our observations and analysis to other MD models of water, which include both more detailed water models with additional sites [21,22] and coarse-grained MD models of water [50].

## References

[1] Koneshan S, Rasaiah J, Lynden-Bell M, Lee S 1998 Solvent structure, dynamics and ion mobility in aqueous solutions at 25°C. *J. Phys. Chem. B* 102, 4193–4204. (doi:10.1021/jp980642x)

[2] Kohagen M, Mason P, Jungwirth P 2014 Accurate description of calcium solvation in concentrated aqueous solutions. *J. Phys. Chem. B* 118, 7902–7909. (doi:10.1021/jp5005693) 24802184 10.1021/jp5005693

[3] Dobramysl U, Rüdiger S, Erban R 2015 Particle-based multiscale modelling of intracellular calcium dynamics. (http://arxiv.org/abs/1504.00146)

[4] Corry B, Kuyucak S, Chung S 2000 Test of continuum theories as models of ion channels. II. Poisson-Nernst-Planck theory versus Brownian dynamics. *Biophys. J.* 78, 2364–2381. (doi:10.1016/S0006-3495(00)76781-6) 10777733 10.1016/S0006-3495(00)76781-6 PMC1300826

[5] Erban R, Chapman SJ, Maini P 2007 A practical guide to stochastic simulations of reaction–diffusion processes, 35 p. (http://arxiv.org/abs/0704.1908)

[6] Leimkuhler B, Matthews C 2015 *Molecular dynamics*. Interdisciplinary Applied Mathematics, no. 39 Berlin, Germany: Springer.

[7] Flegg M, Rüdiger S, Erban R 2013 Diffusive spatio-temporal noise in a first-passage time model for intracellular calcium release. *J. Chem. Phys.* 138, 154103 (doi:10.1063/1.4796417) 23614408 10.1063/1.4796417

[8] Erban R, Chapman SJ 2009 Stochastic modelling of reaction–diffusion processes: algorithms for bimolecular reactions. *Phys. Biol.* 6, 046001 (doi:10.1088/1478-3975/6/4/046001) 19700812 10.1088/1478-3975/6/4/046001

[9] Lipkova J, Zygalakis K, Chapman J, Erban R 2011 Analysis of Brownian dynamics simulations of reversible bimolecular reactions. *SIAM J. Appl. Math.* 71, 714–730. (doi:10.1137/100794213)

[10] Shinohara T, Michikawa T, Enomoto M, Goto J, Iwai M, Matsu-ura T, Yamazaki H, Miyamoto A, Suzuki A, Mikoshiba K 2011 Mechanistic basis of bell-shaped dependence of inositol 1,4,5-trisphosphate receptor gating on cytosolic calcium. *Proc. Natl Acad. Sci. USA* 108, 15 486–15 491. (doi:10.1073/pnas.1101677108) 10.1073/pnas.1101677108 PMC3174635 21876165

[11] Serysheva I 2014 Toward a high-resolution structure of IP_3_R channel. *Cell Calcium* 56, 125–132. (doi:10.1016/j.ceca.2014.08.002) 25159857 10.1016/j.ceca.2014.08.002 PMC4162860

[12] Erban R 2014 From molecular dynamics to Brownian dynamics. *Proc. R. Soc. A* 470, 20140036 (doi:10.1098/rspa.2014.0036) 25002825 10.1098/rspa.2014.0036 PMC4032556

[13] Davtyan A, Dama J, Voth G, Andersen H 2015 Dynamic force matching: a method for constructing dynamical coarse-grained models with realistic time dependence. *J. Chem. Phys.* 142, 154104 (doi:10.1063/1.4917454) 25903863 10.1063/1.4917454

[14] Zwanzig R 1973 Nonlinear generalized Langevin equations. *J. Stat. Phys.* 9, 215–220. (doi:10.1007/BF01008729)

[15] Adelman S 1979 Generalized Langevin theory for many-body problems in chemical dynamics: general formulation and the equivalent harmonic chain representation. *J. Chem. Phys.* 71, 4471–4486. (doi:10.1063/1.438200)

[16] Adelman S 1980 Generalized Langevin theory for many-body problems in chemical dynamics: reactions in liquids. *J. Chem. Phys.* 73, 3145–3158. (doi:10.1063/1.440551)

[17] Zwanzig R 2001 *Nonequilibrium statistical mechanics*. Oxford, UK: Oxford University Press.

[18] Berendsen H, Postma J, Van Gunsteren W, Hermans J 1981 Interaction models for water in relation to protein hydration. In *Intermolecular forces* (ed. B Pullman), pp. 331–342. Dordrecht, The Netherlands: D. Reidel Publishing Company.

[19] Berendsen H, Grigera J, Straatsma T 1987 The missing term in effective pair potentials. *J. Phys. Chem.* 91, 6169–6271. (doi:10.1021/j100308a038)

[20] Jorgensen W, Chandrasekhar J, Madura J, Impey R, Klein M 1983 Comparison of simple potential functions for simulating liquid water. *J. Chem. Phys.* 79, 926–935. (doi:10.1063/1.445869)

[21] Huggins D 2012 Correlations in liquid water for the TIP3P-Ewald, TIP4P-2005, TIP5P-Ewald, and SWM4-NDP models. *J. Chem. Phys.* 136, 064518 (doi:10.1063/1.3683447) 22360206 10.1063/1.3683447 PMC4766739

[22] Mark P, Nilsson L 2001 Structure and dynamics of the TIP3P, SPC, and SPC/E water models at 298 K. *J. Phys. Chem. A* 105, 9954–9960. (doi:10.1021/jp003020w)

[23] Lee SH, Rasaiah JC 1996 Molecular dynamics simulation of ion mobility. 2. Alkali metal and halide ions using the SPC/E model for water at 25°C. *J. Phys. Chem.* 100, 1420–1425. (doi:10.1021/jp953050c)

[24] Andersen H 1983 Rattle: a ‘velocity’ version of the Shake algorithm for molecular dynamics calculations. *J. Comput. Phys.* 52, 24–34. (doi:10.1016/0021-9991(83)90014-1)

[25] Nosé S 1984 A unified formulation of the constant temperature molecular dynamics methods. *J. Chem. Phys.* 81, 511–519. (doi:10.1063/1.447334)

[26] Hoover W 1985 Canonical dynamics: equilibrium phase-space distributions. *Phys. Rev. E* 31, 1695–1697. (doi:10.1103/PhysRevA.31.1695) 10.1103/physreva.31.1695 9895674

[27] Perera L, Essmann U, Berkowitz M 1995 Effect of the treatment of long-range forces on the dynamics of ions in aqueous solutions. *J. Chem. Phys.* 102, 450–456. (doi:10.1063/1.469422)

[28] Nymand T, Linse P 2000 Ewald summation and reaction-field methods for potentials with atomic charges, dipoles and polarizabilities. *J. Chem. Phys.* 112, 6152–6160. (doi:10.1063/1.481216)

[29] Holley R 1971 The motion of a heavy particle in an infinite one dimensional gas of hard spheres. *Z. Wahrscheinlichkeitstheorie Verw. Geb.* 17, 181–219. (doi:10.1007/BF00536757)

[30] Dürr D, Goldstein S, Lebowitz J 1981 A mechanical model of Brownian motion. *Commun. Math. Phys.* 78, 507–530. (doi:10.1007/BF02046762)

[31] Dunkel J, Hänggi P 2006 Relativistic Brownian motion: from a microscopic binary collision model to the Langevin equation. *Phys. Rev. E* 74, 051106 (doi:10.1103/PhysRevE.74.051106) 10.1103/PhysRevE.74.051106 17279876

[32] Mao X 2007 *Stochastic differential equations and applications*. Chichester, UK: Horwood Publishing.

[33] Andrews S, Bray D 2004 Stochastic simulation of chemical reactions with spatial resolution and single molecule detail. *Phys. Biol.* 1, 137–151. (doi:10.1088/1478-3967/1/3/001) 16204833 10.1088/1478-3967/1/3/001

[34] Stiles J, Bartol T 2001 Monte Carlo methods for simulating realistic synaptic microphysiology using MCell. In *Computational neuroscience: realistic modeling for experimentalists* (ed. E Schutter), pp. 87–127. Boca Raton, FL: CRC Press.

[35] van Zon J, ten Wolde P 2005 Green's-function reaction dynamics: a particle-based approach for simulating biochemical networks in time and space. *J. Chem. Phys.* 123, 234910 (doi:10.1063/1.2137716) 16392952 10.1063/1.2137716

[36] Franz B, Flegg M, Chapman J, Erban R 2013 Multiscale reaction-diffusion algorithms: PDE-assisted Brownian dynamics. *SIAM J. Appl. Math.* 73, 1224–1247. (doi:10.1137/120882469)

[37] Opplestrup T, Bulatov V, Donev A, Kalos M, Gilmer G, Sadigh B 2009 First-passage kinetic Monte Carlo method. *Phys. Rev. E* 80, 066701 (doi:10.1103/PhysRevE.80.066701) 10.1103/PhysRevE.80.066701 20365296

[38] Meyer H, Biermann O, Faller R, Reith D, Müller-Plathe F 2000 Coarse graining of nonbonded inter-particle potentials using automatic simplex optimization to fit structural properties. *J. Chem. Phys.* 113, 6264–6275. (doi:10.1063/1.1308542)

[39] Izvekov S, Voth G 2005 A multiscale coarse-graining method for biomolecular systems. *J. Phys. Chem. B* 109, 2469–2473. (doi:10.1021/jp044629q) 16851243 10.1021/jp044629q

[40] Noid W 2013 Perspective: coarse-grained models for biomolecular systems. *J. Chem. Phys.* 139, 090901 (doi:10.1063/1.4818908) 24028092 10.1063/1.4818908

[41] Izvekov S, Voth G 2006 Modeling real dynamics in the coarse-grained representation of condensed phase systems. *J. Chem. Phys.* 125, 151101 (doi:10.1063/1.2360580) 17059230 10.1063/1.2360580

[42] Hijón C, Español P, Vanden-Eijnden E, Delgado-Buscalioni R 2010 Mori-Zwanzig formalism as a practical computational tool. *Farad. Discuss.* 144, 301–322. (doi:10.1039/B902479B) 10.1039/b902479b 20158036

[43] Flegg MB, Chapman SJ, Erban R 2012 The two-regime method for optimizing stochastic reaction-diffusion simulations. *J. R. Soc. Interface* 9, 859–868. (doi:10.1098/rsif.2011.0574) 22012973 10.1098/rsif.2011.0574 PMC3306650

[44] Robinson M, Andrews S, Erban R 2015 Multiscale reaction-diffusion simulations with Smoldyn. *Bioinformatics* 31, 2406–2408. (doi:10.1093/bioinformatics/btv149) 25788627 10.1093/bioinformatics/btv149 PMC4495299

[45] Erban R, Flegg M, Papoian G 2014 Multiscale stochastic reaction-diffusion modelling: application to actin dynamics in filopodia. *Bull. Math. Biol.* 76, 799–818. (doi:10.1007/s11538-013-9844-3) 23640574 10.1007/s11538-013-9844-3

[46] Allen T, Kuyucak S, Chung S 2000 Molecular dynamics estimates of ion diffusion in model hydrophobic and KcsA potassium channels. *Biophys. Chem.* 86, 1–14. (doi:10.1016/S0301-4622(00)00153-8) 11011695 10.1016/s0301-4622(00)00153-8

[47] Saunders M, Voth G 2013 Coarse-graining methods for computational biology. *Annu. Rev. Biophys.* 42, 73–93. (doi:10.1146/annurev-biophys-083012-130348) 23451897 10.1146/annurev-biophys-083012-130348

[48] Praprotnik M, Delle Site L, Kremer K 2008 Multiscale simulation of soft matter: from scale bridging to adaptive resolution. *Annu. Rev. Phys. Chem.* 59, 545–571. (doi:10.1146/annurev.physchem.59.032607.093707) 18062769 10.1146/annurev.physchem.59.032607.093707

[49] Nielsen S, Bulo R, Moore P, Ensing B 2010 Recent progress in adaptive multiscale molecular dynamics simulations of soft matter. *Phys. Chem. Chem. Phys.* 12, 12 401–12 414. (doi:10.1039/c004111d) 10.1039/c004111d 20734007

[50] Praprotnik M, Matysiak S, Delle Site L, Kremer K, Clementi C 2007 Adaptive resolution simulation of liquid water. *J. Phys. Condens. Matter* 19, 292201 (doi:10.1088/0953-8984/19/29/292201) 10.1063/1.2819486 18205455

